# Disrupted relationship between blood glucose and brain dopamine D2/3 receptor binding in patients with first-episode schizophrenia

**DOI:** 10.1016/j.nicl.2021.102813

**Published:** 2021-09-13

**Authors:** U. Sauerzopf, A. Weidenauer, I. Dajic, M. Bauer, L. Bartova, B. Meyer, L. Nics, C. Philippe, S. Pfaff, V. Pichler, M. Mitterhauser, W. Wadsak, M. Hacker, S. Kasper, R. Lanzenberger, L. Pezawas, N. Praschak-Rieder, M. Willeit

**Affiliations:** a Department of Psychiatry and Psychotherapy, Division of General Psychiatry, Medical University of Vienna, Austria; b Department of Clinical Pharmacology, Medical University of Vienna, Austria; c Department of Biomedical Imaging and Image-guided Therapy, Division of Nuclear Medicine, Medical University of Vienna, Austria; d Ludwig-Boltzmann-Institute Applied Diagnostics, Vienna, Austria; e Center for Biomarker Research in Medicine CBmed, Graz, Austria; f Centre for Brain Research, Medical University of Vienna, Austria

**Keywords:** (+)-[^11^C]-PHNO, Glucose, Dopamine, Psychosis

## Abstract

An elemental function of brain dopamine is to coordinate cognitive and motor resources for successful exploitation of environmental energy sources. Dopamine transmission, goal-directed behavior, and glucose homeostasis are altered in schizophrenia patients prior to and after initiation of pharmacological treatment. Thus, we investigated the relationship between blood glucose levels and brain dopamine signaling in drug-naïve patients with first-episode psychosis.

We quantified blood glucose levels and binding of the dopamine D_2/3_ receptor agonist radioligand (+)-[^11^C]-PHNO in 15 medication-naïve patients and 27 healthy volunteers employing positron emission tomography.

Whole-brain voxel-wise linear model analysis identified two clusters of significant interaction between blood glucose levels and diagnosis on (+)-[^11^C]-PHNO binding-potential values. We observed positive relationships between blood glucose levels and binding-potential values in healthy volunteers but negative ones in patients with first episode psychosis in a cluster surviving rigorous multiple testing correction located in the in the right ventral tegmental area. Another cluster of homologous behavior, however at a lower level of statistical significance, comprised the ventral striatum and pallidum. Extracellular dopamine levels are a major determinant of (+)-[^11^C]-PHNO binding in the brain. In line with the concept that increased dopamine signaling occurs when goal-directed behavior is needed for restoring energy supply, our data indicate that in healthy volunteers, extracellular dopamine levels are high when blood glucose levels are low and vice-versa. This relationship is reversed in patients with first-episode psychosis, possibly reflecting an underlying pathogenic alteration that links two seemingly unrelated aspects of the illness: altered dopamine signaling and dysfunctional glucose homeostasis.

## Highlights

• Disturbed glycemic control is an intrinsic feature of schizophrenia.
• The neurotransmitter dopamine has a key role in regulating glucose homeostasis.
• We studied brain dopamine and blood glucose levels in first episode psychosis.
• The relationship between glucose and brain dopamine is altered in schizophrenia.

## 1 Introduction

The mortality gap between patients with schizophrenia (SCZ) and the general population has widened in recent decades (Saha et al., 2007). This excess mortality has been largely attributed to higher rates of cardiovascular disease, as well as to an improvement of health and longevity in the general population that could not be translated to patients with SCZ (Franciosi et al., 2005, Hayes et al., 2017). Second generation antipsychotics (SGA) are effective treatment options for positive and negative symptoms of SCZ. However, metabolic side-effects of SGA may in part be responsible for the higher mortality rates in SCZ (Tiihonen et al., 2009, Chwastiak and Tek, 2009). Many patients with SCZ display insulin resistance under SGA treatment (Takayanagi et al. 2012). A recent meta-analysis ranking antipsychotics according to their metabolic effects shows clearly that the efficacy of antipsychotic drugs is directly related to their propensity for inducing metabolic disturbances (Pillinger et al. 2020). However, SCZ also confers an inherent risk for glucose dysregulation: Patients with first-episode SCZ are prone to abnormal glycemic control, such as performance in an oral glucose tolerance test, also before antipsychotic treatment is initiated, and indices of glycemic control were furthermore found to correlate with the severity of positive symptoms (Perry et al., 2016, Perry et al., 2021, Pillinger et al., 2017, Chen et al., 2013).

Imaging studies using a variety of methods have repeatedly found alterations in subcortical dopamine (DA) functioning in patients with SCZ, and the degree of dopamine dysfunction is related to the severity of positive as well as negative symptoms (Abi-Dargham et al., 2000, Abi-Dargham et al., 2009, Howes et al., 2009, Howes et al., 2012, Laruelle et al., 1999, Breier et al., 1997, Weidenauer et al., 2020).

An important aspect of brain dopamine is its role in mediating goal-oriented behavior. Glucose is the most important energy source for the brain. Thus, when food supply is scarce, mechanisms of glucose homeostasis and brain dopamine transmission must closely interact in attributing salience to perceptional stimuli and in reinforcing patterns of behavior that support an efficient exploitation of environmental energy sources. Disturbances in goal oriented behavior underlie many symptoms of SCZ. A disrupted interaction between brain dopamine signaling and glucose homeostasis thus is a candidate mechanism for better understanding the pathogenesis of SCZ.

Increased dopamine signaling in the basal ganglia has been associated with improved metabolic functioning in healthy subjects (ter Horst et al. 2018). Accordingly, bromocriptine, a dopamine receptor agonist, is a licensed antidiabetic drug in the U.S. and many other countries (Holt et al., 2010). Thus, the high prevalence of dysglycemia in patients with SCZ, present already before antipsychotic treatment is initiated, together with well supported evidence on increased subcortical dopamine signaling, led us to pursue the hypothesis of a loss in the physiological relationship between blood glucose homeostasis and brain dopamine transmission in SCZ (Perry et al., 2021, ter Horst et al., 2018).

In order to test this hypothesis, we investigated the relationship between blood glucose levels and subcortical binding of the dopamine D_2/3_ receptor agonist radioligand (+)-[^11^C]-4-propyl-3,4,4a,5,6,10b-hexahydro-2H-naphtho[1,2-b][1,4]oxazin-9-ol ((+)-[^11^C]-PHNO) in healthy volunteers (HV) and drug-naïve patients with first episode psychosis (FEP) in SCZ or schizophreniform disorder using positron emission tomography (PET). Since (+)-[^11^C]-PHNO, is highly sensitive towards fluctuations in brain dopamine levels, (+)-[^11^C]-PHNO non-displaceable binding potential (BP_ND_) values are to a large part a reflection of extracellular DA levels (Willeit et al., 2008; Ginovart et al. 2006a).

## 2 Materials and methods

### 2.1 Study cohort

A cohort of 41 HV and 29 FEP patients was recruited at the Department of Psychiatry and Psychotherapy, Medical University of Vienna (for details see Weidenauer et al. 2020). Of these 41 HV, seven were part of a test–retest study to establish the PET scanning protocol on site and disregarded for the analysis at-hand. FEP was diagnosed according to DSM-IV employing the criteria for schizophrenia and schizophreniform disorder. Groups were matched for sex and age. Patients were excluded if they had received any antipsychotic treatment within two weeks prior to scanning, if lifetime exposure exceeded 50 mg haloperidol-equivalent, or if they had received any long acting injectable antipsychotic treatment in their life. Overall, three FEP patients had been previously exposed to antipsychotics (Aripiprazole, Olanzapine, and Quetiapine) far below the aforementioned dose limit and were medication-free for at least two months prior to study inclusion. None of the participants had a history of substance use disorders. Current substance use (except nicotine and caffeine) was ruled out by urine drug screen and self-reports. Absence of psychiatric disorders in HV was established using the M.I.N.I. structured interview (Sheehan, 1998). Physical health of all participants was ascertained by routine laboratory testing, physical examination and electrocardiogram. Subjects with a history of traumatic brain injury were excluded from study participation.

This study (Clinical Trial Registry: EUDRACT 2010-019586-29) was approved by the Ethics-Committee of the Medical University of Vienna and Austrian federal regulatory authorities. Forty-one HV and 29 FEP patients were recruited between 2013 and 2017. Patients were recruited at in- and out-patient units of the General Hospital of Vienna. All participants gave written informed consent before entering the study. Patients with FEP were judged as being competent to fully understand scope, risks, and inconveniences of study procedures by treating psychiatric specialists who were not involved in the study.

### 2.2 PET image acquisition

Participants were not required to fast before PET scans but rather to spend the day and hours prior to scanning according to their custom. Subjects were instructed to refrain from drinking alcohol for at least 24 h before PET scanning. If applicable, participants were asked to consume caffeine and nicotine within usual limits on the day of PET scanning.

PET images were acquired on a GE Advance scanner (General Electric Medical Systems, Milwaukee, WI) with a spatial resolution of six mm full-width at half-maximum (FWHM). Emission data were acquired over 90 min after bolus-injection of 302 ± 79 MBq (mean ± SD) (+)-[^11^C]-PHNO. Radiosynthesis was performed as described previously (Rami-Mark et al., 2013, Pfaff et al., 2019). Employing filtered–back projection, images were reconstructed from sinograms to 15 one minute frames and 15 five minute frames. Attenuation correction was performed using matrices acquired immediately before tracer injection in a five minute transmission scan using a rotating ^68^Ge source. All scans were corrected for decay to the time of radioligand injection. T1 and proton density (PD) weighted 3 T magnetic resonance images (MRI) were acquired for PET image co-registration and delineation of regions of interest (ROI).

### 2.3 Blood glucose levels

Blood glucose levels were measured employing a hand-held device (GlucoMen® areo, A. Menarini Diagnostics, Florence, Italy) (Berti et al. 2015) two to five minutes before radioligand injection using whole blood obtained from a peripheral venous catheter.

### 2.4 Image pre-processing and analysis

Images were analyzed using an anatomically unbiased voxel-wise analysis method. Pre-processing of PET images was conducted using AFNI software (Analysis of Functional Neuro-Images, Bethesda, Maryland, USA. https://afni.nimh.nih.gov/). PET images were co-registered to anatomical MRIs using mutual information as cost function. Images were normalized to the Montreal Neurological Institute (MNI) 152 space by normalizing PD weighted MRIs to the MNI template and then applying the combined transformation matrix of the co-registration and normalization to PET images.

Parametric binding-potential (BP_ND_) maps were generated by voxel-wise application of the simplified reference tissue model (SRTM) as implemented in PMOD software (PMOD Technologies Ltd., Zurich, Switzerland. https://www.pmod.com) to PET images (Gunn et al. 1997). BP_ND_ values in pre-defined regions of interest (ROIs) were obtained using the SRTM and ROMI software (Lammertsma and Hume 1996). Time-activity curves (TACs) from a high-binding ROI (ventral striatum; VST) and the reference region (cerebellar cortex avoiding midline structures) were used to facilitate model fitting in the voxel-wise analysis. A ROI comprising the substantia nigra (SN) and the ventral tegmental area (VTA) was drawn manually by placing a spherical ROI over the point of maximal binding identified in the 20th frame of the dynamical PET in the midbrain bilaterally and termed SN/VTA ROI. The globus pallidus ROI was manually delineated on PD weighted MRI scans. GP and SNVTA ROI size and location were controlled by a second rater. Using the SRTM with cerebellum as reference region is a validated method for analyzing (+)-[^11^C]-PHNO binding to dopamine D_2/3_ receptors in the human brain (Ginovart et al., 2007). (+)-[^11^C]-PHNO BP_ND_ values served as primary outcome measure.

### 2.5 Statistical analysis

Linear models were applied to parametric maps for analyzing effects of diagnosis (FEP vs. HV), blood-glucose levels, and their interaction-term on BP_ND_ values at single-voxel level; sex was entered as covariate of no interest. Analyses were performed employing an unbiased whole-brain approach as well as employing high-passing filtering for obtaining a map of reliably estimated BP_ND_ values (0.6 or above) only.

Initially clusters of significant interaction were identified by cluster correction employing an empirical spatial auto-correlation function implemented in AFNI software (3dClustSim using the “−acf” flag). In a second step results were confirmed employing a permutation based clustering tool provided by AFNIs 3dttest++ function using the “−Clustsim” flag which has been shown to yield a lower rate of false-positives as compared to previous methods (Cox et al. 2018; Ganz et al 2021). All cluster simulations were conducted in the aforementioned mask obtained by high-pass filtering. In addition to the rigorous cluster correction, we carried out a Benjamini-Hochberg false-discovery rate (FDR) correction on whole-brain maps.

BP_ND_ values in ROIs comprising the clusters obtained by the anatomically unconstrained parametric maps analysis were analyzed in general linear models employing the „glm“ function implemented in „R“ software (R Foundation for Statistical Computing, Vienna, Austria; https://www.r-project.org). In addition pearson correlations were computed for ROI based data. Group differences in blood glucose levels were tested using Student‘s *t*-test.

## 3 Results

Of the original Cohort of 34 HV in the study group and 29 FEP patients, six HV and eight FEP patients dropped out of the study prior to the relevant examinations. A cohort of 28 HV and 21 FEP patients completed the study. Data of one HV and three FEP patients did not enter analysis because of insufficient quality of PET data due to motion. Blood glucose data at time of scanning were missing for three FEP patients. The cohort of final analysis consisted of 27 HV and 15 FEP patients **(see** Table 1 **and inline** Supplementary Fig. 1**)**.

**Table 1:** Demographic and clinical description of the study population.

	Healthy Volunteers (N = 27)	Patients with First Episode Psychosis (N = 15)	Test statistic	p-value
Sex (females)	13/27	5/15	χ^2^ 0.37	0.55
Age (years)	26.3 (2.6)	26.1 (7)	t: 0.7	0.95
BMI (kg/m^2^)	23.1 (3.4)	22.1 (2.7)	t: 1.0	0.31
Smoker (yes)	10/27	11/15	Χ^2^ 3.73	0.053 .
Blood glucose levels (mmol/l)	4.99 (1.39)	5.68 (1.19)	t: −1.68	0.10
Radiation dose (Mbq)	308 (87)	289 (68)	t: 0.81	0.42
PANSS positive (a.u.)	NA	21.2 (6.6)	NA	NA
PANSS negative (a.u.)	NA	19.1 (5.4)	NA	NA
PANSS general (a.u.)	NA	39.3 (9.3)	NA	NA
PANSS total (a.u.)	NA	79.5 (15.7)	NA	NA

All values provided as mean (standard deviation) or count.

BMI: Body mass index, PANSS: Positive and negative symptom scale

Trend level at p < 0.1

### 3.1 Parametric maps analyses

**Fig. 1:**
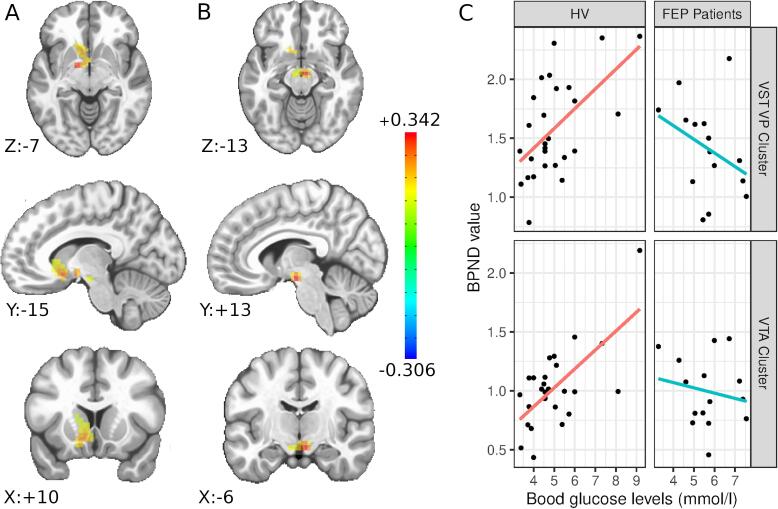
Significant clusters as determined by an anatomically unconstrained analysis in parametric maps. The color bar represents the beta-estimate of the linerar model employed in the parametric map analysis indicating the regional effect size of the blood-glucose: diagnosis interaction term. **A**) Ventral Striatum / Ventral Pallidum (VST/VP) cluster. Peak-voxel at MNI X:+15.6, Y:+3.1, Z:−6.4 located in the left globus pallidus (GP), pars interna. The cluster extends over 3.7 ccm and comprises part of the VST and the VP bilaterally as well as the left GP. **B**) Ventral tegmental area (VTA) cluster. Peak-voxel at MNI X:−6.2, Y:+12.5, Z:−14.9 located in the right VTA. The cluster extends over 1.12 ccm and comprises the VTA bilaterally and parts of the hypothalamus. **C**) For the purpose of visualization we have extracted BP_ND_ values from each cluster and correlated local BP_ND_ values with subject blood glucose values at the time of scanning.

Analysis of parametric maps (sex entered as variable of no interest) revealed two clusters of strong effects of the interaction between blood glucose levels and diagnosis on (+)-[^11^C]-PHNO BP_ND_ values. One cluster of particularly strong effect size was located in a midbrain region corresponding to the ventral tegmental area (VTA; hence: VTA cluster; peak voxel MNI X:−6.2, Y:+12.5, Z:−14.9; defined employing a clustering threshold of p = 0.002; p_cor_ = <0.01 and confirmed by permutation-based clustering at a clustering theshold of p = 0.0005 p_cor_ < 0.05 and FDR correction on whole-brain data q = <0.02). A large cluster was located bilaterally in the ventral striatum and the ventral pallidum (hence VST/VP cluster; peak voxel MNI + 15.6, Y:+ 3.1, Z:−6.4; defined employing a clustering threshold of p = 0.05; p_cor_ = <0.01. Not significant in the permutation based multiple testing correction and FDR; **see** Fig. 1**).**

In order to control for potentially confounding factors, we introduced nuisance variables, possibly impacting dopamine and/or glucose homeostasis (age, smoking status, BMI), into the linear model employed in the parametric maps analysis separately as well as in a full model comprising all these parameters. We employed the rigorous multiple testing correction of the permutation-based cluster correction (3dttest++ using the “−Clustsim” flag) in order to confirm robustness of the clusters discovered in the initial analysis. While the VST/VP cluster was not confirmed by this more conservative method of correction in the initial analysis, the VTA cluster was confirmed significant at the original level of p_cor_ < 0.05 at a clustering threshold of p = 0.0005 after the introduction of each individual nuisance variable into the parsimonious model.

### 3.2 ROI analysis

**Fig. 2:**
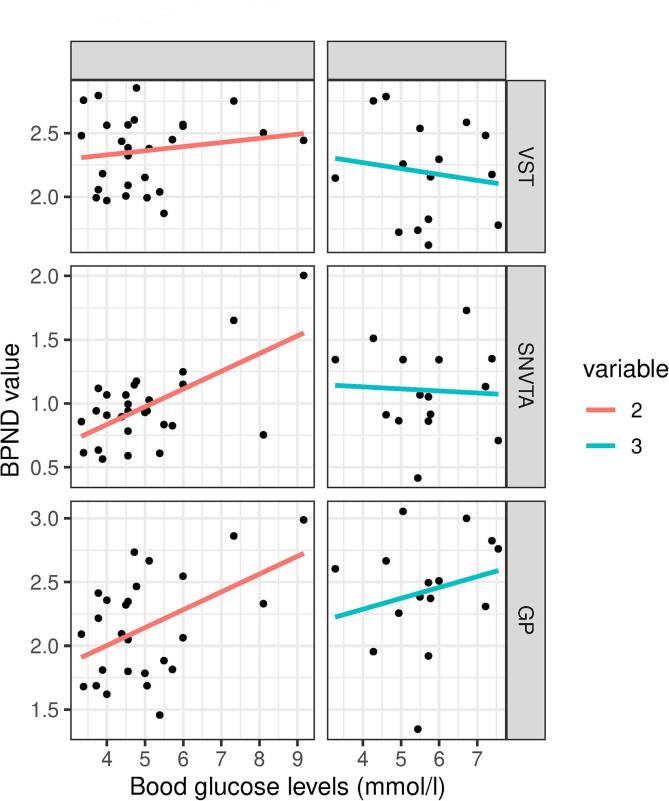
Pearson correlations between blood glucose levels and (+)-[^11^C]-PHNO BPND values in the left ventral Striatum (VST) region of interest (ROI), the right substantia nigra / ventral tegmental area (SN/VTA) ROI, and the globus pallidus (GP) ROI were analyzed in healthy volunteers (HV) and patients with first-episode psychosis (FEP). There are significant correlations between blood glucose levels and BP_ND_ values in HV in the right SN/VTA (p = 0.012, r = 0.48) and the GP (left: p = 0.008, r = 0.50; right: p = 0.012, r = 0.48). No significant correlations were observed in FEP patients.

We have conducted the aforementioned linear model in the SN/VTA ROI, the GP ROI and the VST ROI for each hemisphere. In HVs there were significant pearson correlations between blood glucose levels and BP_ND_ values in the GP ROI bilaterally (left: p = 0.008, r = 0.50; right: p = 0.012, r = 0.48) and the right SN/VTA ROI (p = 0.012, r = 0.48). There were no significant correlations between blood glucose levels and BP_ND_ values in FEP patients. The linear model assessing the relationship between BP_ND_ values, blood glucose levels, diagnosis and the interaction term of glucose and diagnosis yielded significant results only for the right SN/VTA **ROI (see** Table 2 **and** Fig. 2 **and for additional information on lateral differences see inline** Supplementary Fig. 2**)**

**Table 2:** Model characteristics for the right SN/VTA ROI derived model.

Parameter	Beta-coefficient	Regression-coefficient-estimate	standard error	t value	p value
Intercept	0	−1.62	0.97	−1.68	0.1
Glucose (mmol/l)	1.91	0.46	0.18	2.63	0.012 *
Diagnosis (FEP)	1.54	1.04	0.43	2.41	0.021 *
Sex (male)	−0.25	−0.16	0.1	−1.69	0.099 .
Glucose × Diagnosis	−2.38	−0.17	0.08	−2.24	0.031 *

The model investigating the right substantia nigra / ventral tegmental area (SN/VTA) region of interest yielded a p-value of p = 0.006 with an R^2^ of 0.31 and an R^2^ adjusted for the number of parameters of 0.24.

Trend level at p < 0.1;* significant at p < 0.05.

### 3.3 Non-imaging measures

Despite current *meta*-analyses only describe a difference in glycemic control but not in fasting blood glucose levels, a trend towards higher blood glucose levels was observed in FEP patients (HV: 4.99 mmol/l FEP: 5.68 mmol/l. P = 0.087) (Pillinger et al., 2017, Pillinger et al., 2020, Perry et al., 2021). Patients with FEP and HV did not differ in body mass index (p = 0.41) or age (p = 0.95). There was a trend-wise difference in the prevalence of smoking status with numerically higher frequency of tobacco use in patients with FEP (p = 0.053).

## 4 Discussion

In this study, we used PET and the dopamine D_2/3_ receptor agonist radioligand (+)-[^11^C]-PHNO for assessing the relationship between brain dopamine signaling and glucose homeostasis in HV and drug-naïve patients with FEP. We observed strong positive correlations between blood glucose levels and subcortical (+)-[^11^C]-PHNO BP_ND_ values in HV. Since (+)-[^11^C]-PHNO BP_ND_ values are primarily reflecting brain extracellular dopamine levels, our data indicate that in HV, extracellular dopamine levels are high when blood glucose levels are low and vice-versa (Caravaggio et al. 2015a). Although not allowing for causal inference, our data, for the first time to our knowledge, show that there exists a physiological relationship between blood glucose levels and brain dopamine signaling. According to our data, this relationship is perturbed – if not reversed – in drug-free patients with FEP.

### 4.1 Interpretation of findings

We initially described two clusters of interaction between blood glucose levels and the (+)-[^11^C]-PHNO BP_ND_ values in the VTA and the VST/VP. While the VTA cluster has proven to be robust to rigorous multiple testing correction as well as the introduction of nuisance variables into the model, the VST/VP cluster did not survive these more conservative corrections. Nevertheless, the analogous behavior of these two clusters make the assumption reasonable that both dopaminergic cell bodies in the VTA as well as its projection areas in VST and VP show the same physiological interaction of dopamine and blood glucose levels, an interaction that according to our results, is disrupted in patients with FEP: There was a positive relationship between blood glucose levels and (+)-[^11^C]-PHNO BP_ND_ values in HV but not in patients with FEP. A highly significant effect of diagnosis on the correlation between blood glucose and BP_ND_ values was revealed by the linear model analysis. The difference in the relationship of blood glucose and BP_ND_ values between HV and FEP patients was not only strong enough to offset the physiological positive correlation but resulted in a net negative correlation in FEP patients. The ROI analysis yielded analogous results with the effect being most pronounced in the right SN/VTA ROI. This is in line with the parametric maps analysis. However, the lack of association in the VST ROI as well as the generally lower levels of significance may be attributed to the heterogeneous behavior of the VST and GP which notably display regional specialization better captured by a voxel-wise approach In summary, for the first time, we show a differential relationship between blood glucose and D_2/3_ receptor availability in HV and medication naïve patients with FEP.

Investigating D_2/3_ receptors availability in medication naïve FEP patients provides the unique possibility to study disease-specific phenotypes unaltered by antipsychotic medication. (+)-[^11^C]-PHNO is a D_2/3_ receptor agonist radioligand. It is sensitive towards competition with extracellular dopamine and allows for reliable estimation of binding potentials not only in neostriatal areas and the pallidum but also in the SN/VTA (Willeit et al., 2008, Ginovart et al., 2006). Due to its sensitivity towards competition with dopamine, (+)-[^11^C]-PHNO BP_ND_ values are, in large part, a reflection of extracellular dopamine levels with an indirect proportional relationship (Caravaggio et al. 2015a). Nevertheless, the relative contribution of receptor quantity, affinity, or competition with extracellular dopamine cannot be disentangled by the experiments conducted. Our data corroborate evidence that metabolic dysfunction in schizophrenic psychosis pre-dates exposition towards antipsychotic drugs and occurs early in the course of the disorder. Most importantly, our data show that compared to the general population, the interplay between dopamine signaling and blood glucose follows a different pattern in FEP.

### 4.2 Dopamine and insulin signaling

The relationship between severity of positive symptoms and insulin resistance in medication naïve FEP patients further emphasizes the pathophysiological relevance of perturbed glucose homeostasis in SCZ (Chen et al. 2013). In humans and mice alike, glucose homeostasis is highly dependent on the striatal dopaminergic tone, with dopamine depletion reducing peripheral insulin sensitivity and hyper-dopaminergia increasing insulin sensitivity (ter Horst et al. 2018). This is in line with the findings of our study, where in HV, higher extracellular dopamine levels, as approximated by lower BP_ND_ values, were associated with lower blood glucose. Dopamine and insulin signaling overlap in their second messenger cascade and influence each other on an intracellular level. D_2/3_ receptors do not exclusively rely on G-protein coupled pathways in terms of intracellular signal transduction. The β-arrestin pathway is particularly relevant for non-immediate dopamine receptor response (Beaulieu et al., 2011). Downstream of dopamine D_2/3_ receptors, β-arrestin signaling is dependent on AKT (also known as Protein Kinase B) and mTOR (mammalian target of rapamycine), where dopamine D_2/3_ receptor activation inhibits AKT phosphorylation which in turn decreases activation of mTOR. Phosphorylated AKT induces translocation of dopamine transporters on the cell surface, in turn reducing the dopaminergic tone (Nash 2017). Insulin may be considered a counterpart to dopamine signaling in the AKT/mTOR cascade as insulin receptors are activators of AKT. It appears likely that increased insulin signaling is either an adaptation to a primarily increased dopaminergic tone, leading to consecutive desensitization of the insulin receptor, or that hyper-dopaminergia is secondary to a perturbation in the molecular machinery fine-tuning insulin and dopamine signaling. The molecular intricacies of the dopamine-insulin interplay in SCZ remain yet to be fully explored. Dopamine is a phylogenetically old neurotransmitter that is generally linked to the adaptation of behavior in relation to rewarding stimuli, most noteworthy food. In *Caenorhabdis elegans,* dopamine modulates movement to maximize time spent in the presence of a food source (Sawin et al. 2000). In mammals, dopamine signaling is tightly linked to prediction errors commonly associated with food-reward but also other rewarding or averse stimuli (Schultz et al 1997). Considering that disturbances in volition and goal oriented behavior are core negative symptoms of SCZ, the molecular link between dopamine and glucose homeostasis appears a natural target for research into the pathogenesis of SCZ. Interestingly, olanzapine, one of the most efficacious antipsychotics known for its severe metabolic side-effects, is an activator of mTOR (Schmidt et al. 2013). A steep increase in insulin resistance going along with an increase in BMI, has been observed in healthy volunteers after ten days of olanzapine intake (Sacher et al. 2008). This implies that metabolic side-effects of current antipsychotics may indeed be more than a nuisance but be tightly linked their anti-psychotic efficacy (Pillinger et al. 2020). Understanding the delicate balance of insulin and dopamine signaling in the brain and thus, the relevance of the mTOR/AKT pathway, may yet allow us to disentangle antipsychotic effects and metabolic side effects in future drug development.

### 4.3 Limitations

First and foremost, interpretation of the data obtained from the VST/VP cluster has been taken with a grain of salt as it did not prove robust towards most rigorous multiple testing corrections. The VTA cluster however, which behaves analogous to the VST/VP cluster, was proved significant by all tests employed as well as confirmation by the ROI analysis. The critique on some older, less conservative cluster correction methods is based on observations on false-positive rates in functional MRI studies where noise distribution did not correspond to previous assumptions (Eklund et al., 2016, Cox et al., 2017). There is some evidence that the problem also occurs in PET data (Ganz et al. 2021) so that we conducted a permutation based clustering approach which has been shown to effectively reduce false positives below five percent in the parametric maps analysis (Ganz et al., 2021, Cox et al., 2017).

In order to provide a holistic view, we also provide data from the less robust VST/VP cluster. Our study is further limited by a lack of control for food intake prior to scanning. While this is a naturalistic design, the relationship between blood glucose levels and (+)-[^11^C]-PHNO BP_ND_ values could be confounded by different nutritional patterns in HV and patients with FEP. In previous studies, striatal binding of the D_2/3_ receptor radioligand [^11^C]raclopride was found to be negatively correlated with BMI, while binding of (+)-[^11^C]-PHNO was found to correlate positively with BMI (Volkow et al., 2008, Wang et al., 2001, Caravaggio et al., 2015b, Gaiser et al., 2016). These seemingly contradicting results become understandable when considering the differing binding characteristics of the two radioligands: The D_2/3_ antagonist [^11^C]raclopride is mostly measuring the D_2/3_ receptor density irrespective of the receptor affinity state; in contrast, binding of the agonist (+)-[^11^C]-PHNO is largely determined by extracellular dopamine levels being more susceptible to fluctuating on endogenous dopamine concentrations and preferential binding to D_2/3_ receptors in a high affinity state. Thus, notwithstanding slight differences in the physiological processes captured by the two methods, this supports the interpretation of our data, as low binding of [^11^C]raclopride (low receptor availability), and high binding of (+)-[^11^C]-PHNO (low levels of extracellular dopamine) would reflect a decrease in dopamine transmission, and vice versa. In our data, adding BMI as a covariate in the parametric maps analysis did not impact the results. This may be due to the narrow range of BMI values in our study population. There was a trend towards a higher rate of smoking in patients with FEP. This is in-line with previous investigations, where patients with SCZ consistently display higher rates of smoking than healthy controls (Kelly and McCreadie 2000). The acute effects of cigarette smoking on glucose tolerance in healthy volunteers remains disputed, but may be considered negligible for our purposes (Nilsson et al., 1995, Sakai et al., 2006, Frati et al., 1996). In the parametric maps analysis, there was no significant effect of smoking on BP_ND_ levels. Introduction of the variable did not impact the overall interaction of blood glucose levels with diagnosis in either region. Similarly, complementary analyses correcting for age did not reveal a significant effect on the observed interaction. The sample size of our study, albeit small in absolute terms, is within common ranges for PET studies and sufficient to yield highly significant results (Laruelle et al., 1999, Breier et al., 1997, Caravaggio et al., 2015b). There were relatively fewer FEP patients than HV in our study. This leads to somewhat lower power in detecting effects within the FEP group. It has, however, been shown in the past, that not all cases of SCZ display the same neurochemical patterns with respect to dopamine neurotransmission, so that a selection bias within a small sample of FEP patients could limit inference from our data to a general population of patients with SCZ (Brugger et al. 2020). While the (+)-[^11^C]-PHNO BP_ND_ value is tightly correlated to the availability of brain extracellular dopamine, it remains a surrogate marker for actual dopamine levels. Blood glucose levels were obtained employing a hand-held device generally delivering less accurate results than laboratory measurements. This introduces noise into the data and might have led to lower power in detecting blood-glucose dependent effects. Moreover, no measurements of insulin or insulin resistance were taken in this study, so that our claims remain limited to the association between blood glucose levels and dopamine signaling. Further studies are warranted investigating parameters more directly involved in the proposed pathways, such as insulin and insulin resistance. However, estimates for regression coefficients of the diagnosis-glucose interaction obtained in the general linear model were comparable between regions and proved to be robust against the effects of nuisance variables. Due to the limited spatial resolution of our PET scanning system, we were unable to definitely separate SN from the VTA. However, according to MNI coordinates, the point of maximum interaction was located in the VTA rather than the SN. Stress mediated dopamine release in the SN/VTA area has previously been shown to be grossly different in HV and patients with FEP – more so than in striatal areas proper, highlighting the pathophysiological importance of midbrain dopaminergic areas in SCZ (Tseng et al. 2018).^.^ The VST, an important projection area of VTA dopaminergic neurons, has been associated with cue dependent learning as well as the pathophysiology of psychosis (Kapur, 2003, Weidenauer et al., 2020).

## 5 Conclusion

In summary, our data give strong and robust indication of a disease-specific phenotype in SCZ, as the physiological interdependency of dopamine signaling and glucose homeostasis appears to be disturbed in medication naïve patients with FEP. Perturbation of the dopamine-insulin interplay in the brain may be a fundamental aspect of schizophrenic psychosis. It appears as though dopamine, physiologically necessary for movement and volition and typically associated with a catabolic state, and insulin signaling, typically excreted after the ingestion of food and associated with rest and an anabolic state, do not interact in a canonic way in FEP patients. The physiological relationship between dopamine signaling and glucose homeostasis is perturbed in FEP patients, possibly reflecting an underlying pathogenic alteration linking these seemingly unrelated aspects of schizophrenic psychosis.

## CRediT authorship contribution statement

**Ulrich Sauerzopf:** Data curation, Formal analysis, Investigation, Validation, Visualization, Writing - original draft. **Ana Weidenauer:** Data curation, Formal analysis, Investigation, Project administration, Validation, Writing - review & editing. **Irena Dajic:** Data curation, Formal analysis, Writing - review & editing. **Martin Bauer:** Conceptualization, Investigation, Methodology, Project administration, Resources, Validation, Writing - review & editing. **Lucie Bartova:** Data curation, Investigation, Validation, Project administration. **Bernhard Meyer:** Data curation, Investigation, Software, Validation. **Lukas Nics:** Data curation, Investigation, Resources, Validation, Writing - review & editing. **Cecile Philippe:** Data curation, Investigation, Resources, Validation, Writing - review & editing. **Sarah Pfaff:** Data curation, Investigation, Resources, Validation, Writing - review & editing. **Verena Pichler:** Data curation, Investigation, Resources, Validation, Writing - review & editing. **Markus M. Mitterhauser:** Methodology, Resources, Validation, Supervision, Writing - review & editing. **Wolfgang Wadsak:** Conceptualization, Project administration, Methodology, Resources, Validation, Supervision, Writing - review & editing. **Marcus Hacker:** Project administration, Resources, Supervision, Writing - review & editing. **Siegfried Kasper:** Resources, Supervision, Writing - review & editing. **Rupert Lanzenberger:** Resources, Supervision, Writing - review & editing. **Lukas Pezawas:** Resources, Supervision, Validation, Writing - review & editing. **Nicole Praschak-Rieder:** Conceptualization, Data curation, Supervision, Validation, Writing - review & editing. **Matthäus Willeit:** Conceptualization, Data curation, Formal analysis, Funding acquisition, Investigation, Methodology, Project administration, Resources, Supervision, Validation, Writing - review & editing.

## Funding

This project was funded by the Vienna Science and Technology Fund (WWTF, Grant number: CS15-033), the Austrian Science Fund (FWF, Grant number: P23585-B09), the Anniversary Fund of the Austrian National Bank (Grant number. 16723), the Medical Scientific Fund of the Mayor of the City of Vienna (Grant number 15189), all granted to M.W.

## Declaration of Competing Interest

Without relevance to this work, M. Willeit declares to having received speaker honoraria and consulting fees from Janssen-Cilag Pharma GmbH, Austria. Without relevance to this work, W. Wadsak declares to havingreceived speaker honoraria from GE Healthcare, research grants from IpsenPharma, Eckert-Ziegler AG, Scintomics and ITG. WW is a part time eployee ofCBmed Ltd (Center for Biomarker Research in Medicine, Graz, Austria). and is a member of advisory boards of Amgen, Chieri and Sanofi-Aventis. M. Hacker received consulting fees and/or honoraria from Bayer Healthcare BMS, Eli Lilly, EZAG, GE Healthcare, Ipsen, ITM, Janssen, Roche, Siemens Healthineers. R. Lanzenberger received travel grants and/or conference speaker honoraria within the last three years from Bruker BioSpin MR, Heel, and support from Siemens Healthcare regarding clinical research using PET/MR. He is a shareholder of the start-up company BM Health GmbH since 2019. S. Kasper received grants/research support, consulting fees and/or honoraria within the last three years from Angelini, AOP OrphanPharmaceuticals AG, Celegne GmbH, Eli Lilly, Janssen-Cilag Pharma GmbH, KRKA-Pharma, Lundbeck A/S, Mundipharma, Neuraxpharm, Pfizer, Sanofi, Schwabe, Servier, Shire, Sumitomo Dainippon Pharma Co. Ltd. and Takeda. U. Sauerzopf, A. Weidenauer, I. Dajic, M. Bauer, L. Bartova, B. Meyer, L. Nics, C. Philippe, S. Pfaff, V. Pichler, M. Mitterhauser, L. Pezawas and N. Praschak-Rieder have no conflict of interest to declare.
