# Supplementary material for: Disrupted relationship between blood glucose and brain dopamine D2/3 receptor binding in patients with first-episode schizophrenia
Source: Neuroimage Clin. 2021 Sep 13;32:102813. doi: 10.1016/j.nicl.2021.102813 (PMC8455866; doi:10.1016/j.nicl.2021.102813)
Supplement: Supplementary data 1 [file mmc1.docx]

**
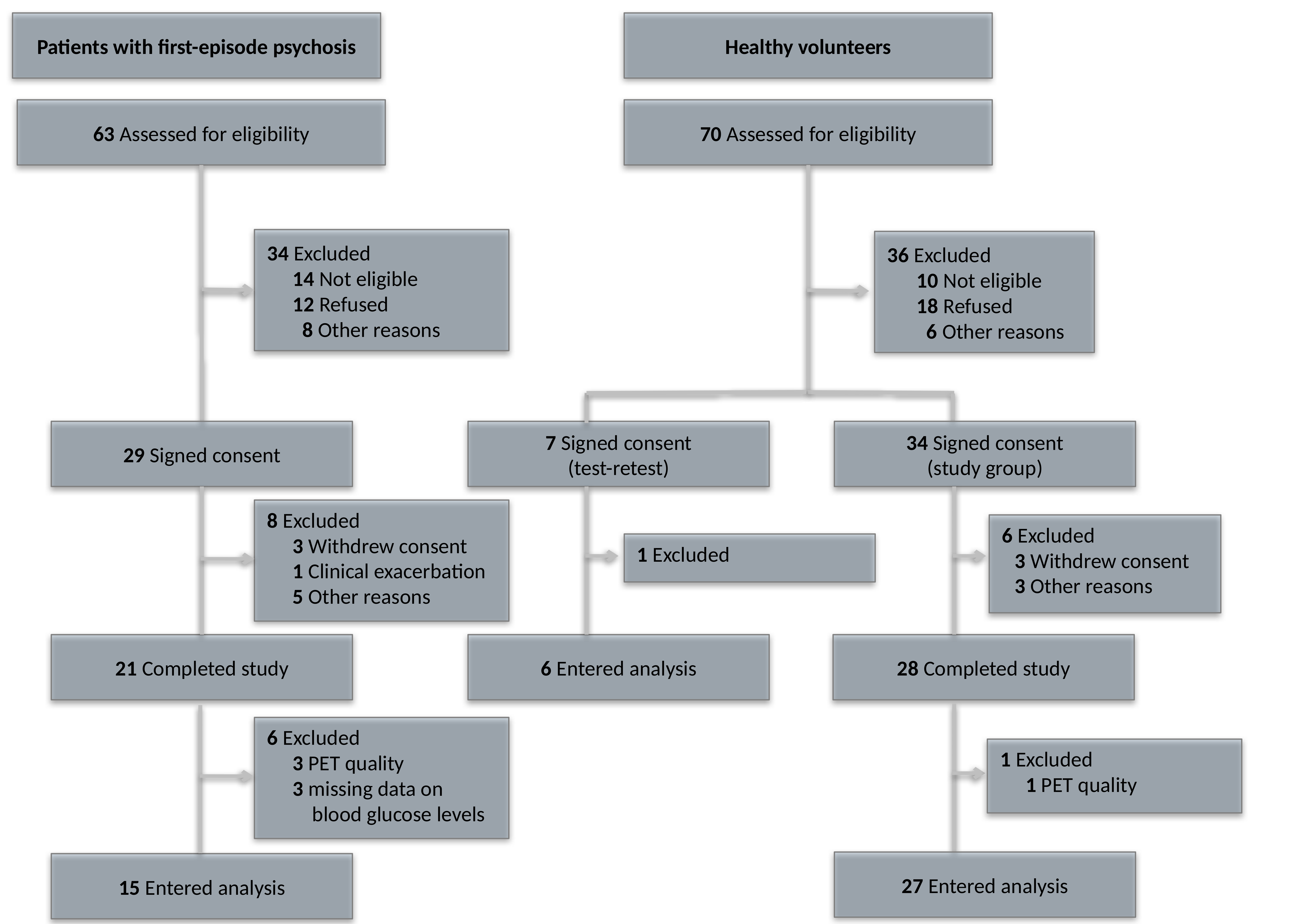
**

**Inline supplementary Figure 1:** *Study flow chart.*


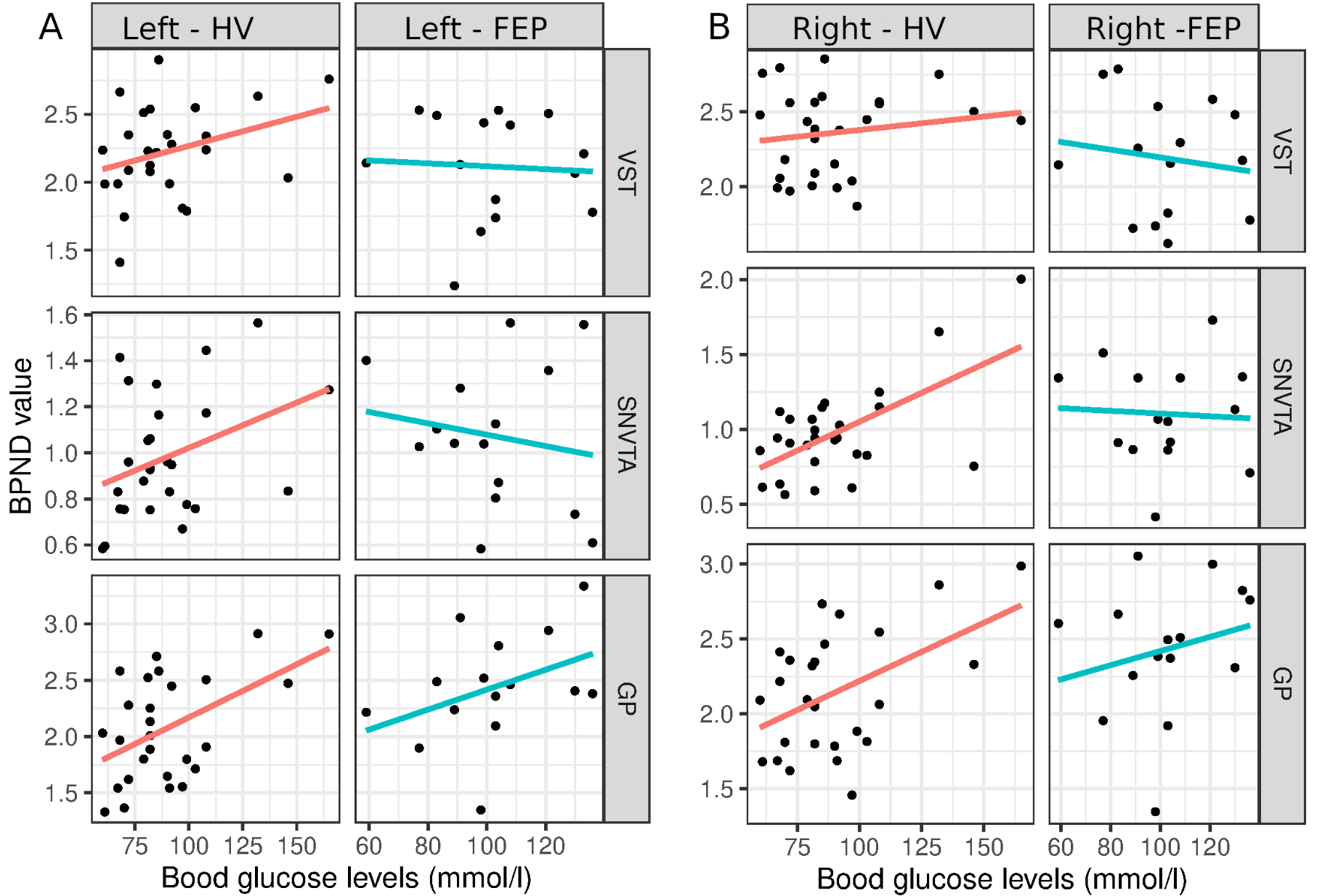


***Inline supplementary Figure 2:*** *Pearson correlations between blood glucose levels and (+)-[^11^C]-PHNO BP_ND_ values in the substantia nigra / ventral tegmental area region of interest (ROI), the ventral striatum (VST) ROI, and the globus pallidus ROI for both hemispheres. Panel A (left) displays the left hemisphere, panel B (right) the right hemisphere. There was a strong correlation in the SN/VTA in healthy volunteers on the right hemisphere (left p= 0.058, r= 0.37; right p= 0.00068, r= 0.61) that was not observed in first episode psychosis (FEP) patients (left: p= 0.55, r= -0.17; right: p= 0.84, r= -0.06). Similarly in the GP positive correlations were observed in HV (HV left: p= 0.008, r: 0.50; HV right p= 0.012 r= 0.48) but not in FEP patients (FEP left: p= 0.39, r= 0.15; FEP right: p= 0.41, r= 0.22). In the VST no significant correlations were observed in either HV (HV left: p= 0.10, r= 0.32; HV right: p= 0.44, r= 0.16) or FEP patients (FEP left: p= 0.83, r= -0.06); FEP right: p= 0.61, r= -0.14).*
